# The Molecular Mechanisms Associated with Aerobic Exercise-Induced Cardiac Regeneration

**DOI:** 10.3390/biom11010019

**Published:** 2020-12-27

**Authors:** Bing Bo, Yang Zhou, Qingyun Zheng, Guandong Wang, Ke Zhou, Jianshe Wei

**Affiliations:** 1 School of Physical Education, Henan University, Kaifeng 475001, Henan, China; bobing@henu.edu.cn (B.B.); 104753190403@henu.edu.cn (Y.Z.); zhengqy@henu.edu.cn (Q.Z.); wangguandong@henu.edu.cn (G.W.); 10180055@vip.henu.edu.cn (K.Z.); 2 Sports Reform and Development Research Center, Henan University, Kaifeng 475001, Henan, China; 3 School of Life Sciences, Henan University, Kaifeng 475001, Henan, China

**Keywords:** cardiomyocyte, regeneration, aerobic exercise, signaling pathways, molecular mechanism

## Abstract

The leading cause of heart failure is cardiomyopathy and damage to the cardiomyocytes. Adult mammalian cardiomyocytes have the ability to regenerate, but this cannot wholly compensate for myocardial cell loss after myocardial injury. Studies have shown that exercise has a regulatory role in the activation and promotion of regeneration of healthy and injured adult cardiomyocytes. However, current research on the effects of aerobic exercise in myocardial regeneration is not comprehensive. This review discusses the relationships between aerobic exercise and the regeneration of cardiomyocytes with respect to complex molecular and cellular mechanisms, paracrine factors, transcriptional factors, signaling pathways, and microRNAs that induce cardiac regeneration. The topics discussed herein provide a knowledge base for physical activity-induced cardiomyocyte regeneration, in which exercise enhances overall heart function and improves the efficacy of cardiac rehabilitation.

## 1. Introduction

Heart failure (HF) is the leading cause of human morbidity and mortality in the world [1]. HF is a clinical syndrome caused by myocardial structural and/or functional abnormality (e.g., abnormalities of the valves, pericardium, endocardium, arrhythmia, and conduction), resulting in a reduced cardiac output and/or elevated intracardiac pressures at rest or during stress [2]. Cardiomyopathies are an important cause of HF with decreased ejection fraction. Dilated cardiomyopathies (DCM) induce HF with reduced ejection fraction (HFrEF), while hypertrophic cardiomyopathies (HCM) trigger HF with preserved ejection (HFpEF) [3,4]. About 50% of HF patients die within five years after the initial diagnosis, which exceeds most cancer [5]. HF encompasses various diseases affecting the heart and can lead to fatal cardiac events [1,6]. Death and loss of adult cardiomyocytes and supporting tissues is a primary driver of pathological ventricular remodeling, which ultimately leads to HF. Except for heart transplant surgery, there is currently a lack of effective treatment to supplement injured myocardial cells within the myocardium.

Previous investigations have demonstrated that mammalian hearts have a limited ability to regenerate following myocardial injury. Existing cardiomyocytes are the primary source of regeneration rather than endogenous progenitor cells [7,8,9]. Several DNA isotope quantitative studies have shown that cardiac regeneration is highest in early childhood and reduces gradually to less than 1% in adulthood [10,11]. Stimulating the physiological cardiac growth signaling cascade and preventing cardiomyocyte loss is an effective way to maintain cardiac function in the case of myocardial overload or damage. Theoretically, the ideal treatment strategy for HF is to replace dead cardiomyocytes with neonatal cardiomyocytes, which electro-chemically couple and communicate with healthy tissue to restore the normal physiological function of damaged myocardium [12]. However, current clinical treatment options remain focused on myocardial rescue rather than on replacement [13].

For centuries, the benefits of regular exercise to improve cardiac function and treat heart disease have been recognized [14,15]. Cardiovascular adaptation due to many years of exercise training includes a sustained increase in cardiac output and the dilation of cardiac dimensions by 10–20%. Chronic exercise training preserves the systolic function of the left ventricle and minimizes the impacts of myocardial infarction (MI) in athletes [16]. Exercise may also attenuate the pathological process and lay the foundation for cardiac remodeling. Routine exercise changes the individual cardiovascular profile and reduces the risk of MI by ~50% for coronary artery disease [17]. Besides improving cardiac function in physiological and pathological settings, regular exercise also increases myocardial mass, likely due to the increase in myocardial cell size [18]. From a cellular-mechanistic perspective, several investigations have found that transcription factors related to cell proliferation and differentiation dramatically increase the expression of exercised hearts [19,20]. These studies suggest that exercise may impact the cell cycle phase of existing cardiomyocytes and promote their re-entry into the cell cycle. Additionally, the reduction of metabolic regulators expression, adipose triglyceride lipase (ATGL/bmm) and transcriptional cofactor PGC-1 is associated with heart dysfunction and lipotoxic cardiomyopathy with elevated systemic H3K27 trimethylation by high-fat-diet (HFD) [21]. A rapid shift from anaerobic glycolysis to fatty acid β-oxidation in myocardium after birth is thought to play a role in cardiomyocyte cell-cycle arrest by induction of DNA damage response pathway in mammalian heart [22]. Inhibiting fatty-acid utilization by pyruvate dehydrogenase kinase 4 (PDK4) delete resulted in pyruvate dehydrogenase activity increase, cardiomyocyte size reduction, DNA damage relief and an increase in cardiomyocyte proliferation [22]. Exercise also could regulate glycolysis [23] and fatty acid metabolism [24] to promote cardiac growth. Numerous studies have indicated that exercise regulates complex molecular pathways and cellular mechanisms (signaling pathways, paracrine factors, transcriptional factors, microRNAs (miRNAs)) that induce cardiac regeneration [20,25,26]. An in-depth understanding of the development of new cardiomyocytes caused by exercise will enable us to develop new therapies for heart disease.

## 2. Regeneration Capacity of the Heart Differs by Species and Life Stage

The adult mammalian heart is a post-mitotic organ and switches abruptly from hyperplasia to hypertrophy during development and growth after birth. However, an increasing number of studies have gradually raised the possibility of triggering the heart to switch back to unlocking the cardiomyocytes to regenerate new myocardium in adults [27]. Cardiac regeneration differs among species and life stages. For example, cardiomyocytes from frogs, newts, and zebrafish maintain the ability to proliferate and differentiate throughout life [28,29]. In adult zebrafish, there is a significant increase in cycling myocytes labeled with 5-bromo-2′-deoxyuridine (BrdU) following the surgical resection of ~20% of the ventricle. After partial excision of the adult zebrafish or amphibian heart, the increase of new cardiomyocytes nearly restores the full function and structure of the ventricle. Others have shown that this degree of regeneration is insufficient in response to more severe injuries [29]. Unlike zebrafish and amphibians, myocardial regeneration is limited in adult mammalian hearts. Porrello et al. found that newborn mice have sufficient regeneration capacity in cardiomyocytes to repair injuries [30]. However, the mice lose this regenerative capacity after postnatal day seven [31]. A similar phenomenon occurs in the human neonatal MI hearts, which suggests that human newborns also have the ability to regenerate cardiomyocytes [32].

The studies mentioned above have identified a transition period for myocardial regenerative capacity after birth in mammals, which is related to the period from which cardiomyocytes withdraw from the cell cycle. Bergmann et al. used a radiocarbon (^14^C) method to estimate that up to 45% of cardiomyocytes interchange during a person’s whole lifetime [10,11]. However, the extent of this recovery is incapable of supplementing enough cardiomyocytes to reconstruct the physiological function in the injured myocardium [33,34].

The heart tissue of adult mammals lacks a special source of stem cells that can produce cardiomyocytes [9,35], and non-cardiomyocytes do not contribute to any new cardiomyocytes in homeostatic or stress conditions in the adult heart [9]. Cardiomyocytes in newborns [9,31] and in the injured myocardium [8,9] are mainly derived from pre-existing cardiomyocytes [7]. Mature cardiomyocytes can re-enter the cell cycle and form new cells in post-MI cardiac tissues through dedifferentiation, proliferation, and redifferentiation [36].

## 3. Effects of Aerobic Exercise on the Heart

Chronic exercise training repeatedly increases oxygen delivery to working muscles and other tissues, resulting in physiological changes within the cardiopulmonary and skeletal muscle systems. Cardiac growth caused by exercise can increase myocardial contractility and reduce myocardial ischemia in the mammalian heart [25,37]. However, it remains unclear if there is a dose-dependent response to exercise duration and/or intensity. In the following section, we discuss the impacts of aerobic exercise on cardiac regeneration and explore its effects on cardiomyocyte structure and function.

Based on muscle metabolism, aerobic exercise is a moderate-to-high intensity dynamic exercise that uses large muscle groups continuously and is maintained with rhythmic physical activity [38,39]. Muscle groups obtain energy from carbohydrates, fatty acids, and amino acids when they are activated. Their main form of energy is adenosine triphosphate (ATP). Aerobic exercise includes medium-to-long distance running/jogging, swimming, cycling, dancing, hiking, and walking. Aerobic capacity is the ability of the cardiopulmonary system to provide oxygen to tissues and the ability of the skeletal muscle to utilize oxygen [40,41]. The measurement criterion for aerobic capacity is the maximum rate of oxygen consumption (VO_2_ max), which can be measured during incremental exercise [42].

**Figure 1:**
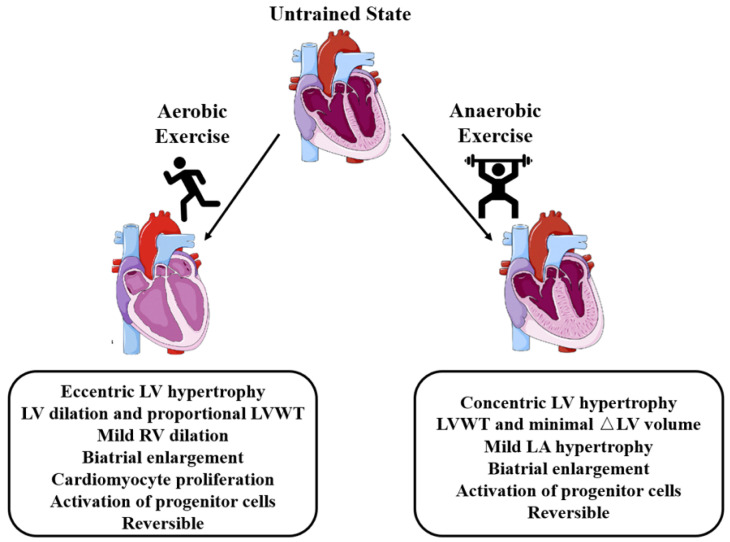
Schematic of exercise-induced cardiac hypertrophy. Aerobic and anaerobic exercise elicits physiological cardiac remodeling. Hypertrophy activation is principally eccentric for aerobic but concentric for anaerobic exercise. LA, left atrium; LV, left ventricle; LVWT, left ventricular wall thickness; RA, right atrium; RV, right ventricle.

Cardiac reactions to aerobic exercise are directly related to oxygen utilization for ATP production in skeletal muscle. Low-intensity physical activity increases oxygen consumption up to ~10 times, but the oxygen flow through muscles is up to ~100 times [43]. At the initial stage of aerobic exercise, the Fick equation defines the relationship between heart rate, ejection fraction, and cardiac output [44,45]. Prolonged isotonic or dynamic aerobic exercise enhances ventricles (left hypertrophy but right dilation) and results in biatrial enlargement (Figure 1). Although the addition of cardiac sarcomeres was thought to be the main reason for this form of hypertrophy, recent studies have shown that exercise can activate cardiac regeneration genes [20,46].

Long-term aerobic exercise can cause an increase in heart mass, known as physiological cardiac hypertrophy, which is different from pathological cardiac hypertrophy in molecular function and tissue morphology [47]. Cardiac remodeling induced by aerobic exercise is combined with an increase in ventricular volume and the number of cardiomyocytes, which is a physiological adaptation via cardiomyogenesis in the mammalian heart. Recent data collected from wearable devices shows that higher-intensity activity and a higher volume of physical activity lowers future health risks and mortality [48]. However, the intensity and frequency of aerobic exercise need to be further evaluated, and exercise plans should be developed according to the individual’s physiological and pathological condition [49].

In addition, from the perspective of oxygen metabolism, anaerobic exercise is also important. In the absence of oxygen, anaerobic exercise (intense physical activity within a short duration) is powered by energy in contracting muscles provided by the phosphate and glycolysis pathways, which causes the accumulation of lactic acid [40]. High-intensity exercise that recruits the fast-twitch muscles include sprinting and powerlifting and may involve transient bursts of increased peripheral vascular resistance without effects on cardiac output. These types of activities are associated with mild concentric hypertrophy with a slightly enlarged left atrium (Figure 1). High-intensity interval training (HIIT) involves repeated intervals of high-intensity activity with intermittent or active low-intensity recovery intervals. The reason why HIIT has attracted widespread attention is because data have suggested that it can improve cardiovascular and metabolic function of healthy people and people with chronic diseases. The application of a HIIT exercise program can increase VO_2_ max [50] and decrease the incidence of major adverse events in patients with cardiovascular disease [51].

## 4. Molecular Mechanisms Related to Aerobic Exercise-Induced Cardiac Regeneration

**Figure 2:**
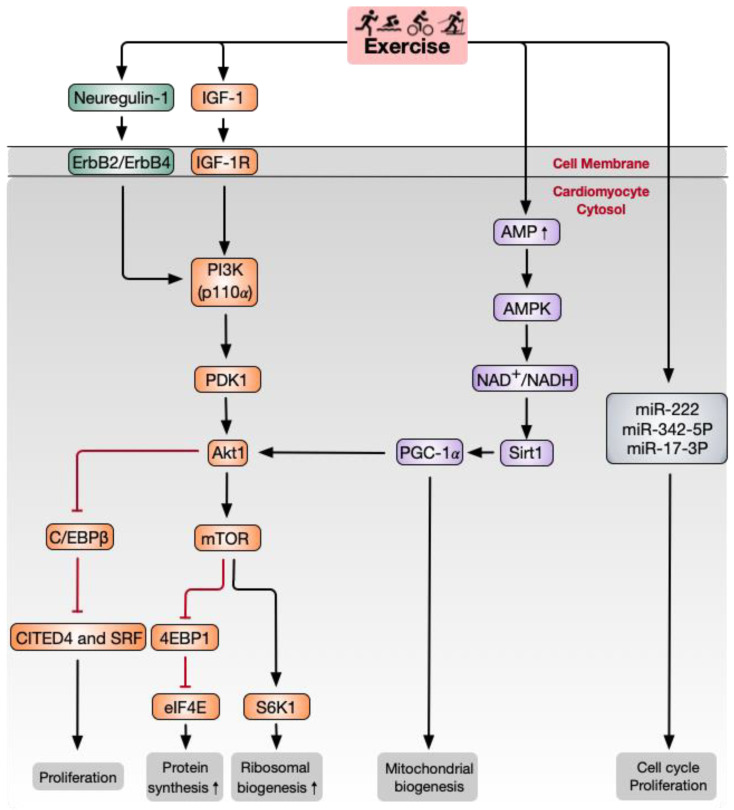
Schematic of major signaling pathways and other factors involved in aerobic exercise-induced cardiomyocyte regeneration. IGF-1 and neuregulin-1 activate PI3K and downstream Akt signaling pathways. Akt activates 4EBP1 and S6K1, downstream signaling molecules of mTOR, which act as crucial factors governing protein biosynthesis in the process of cardiac growth. C/EBPβ and downstream CITED4 are activated by Akt1 and are responsible for cardiomyocyte proliferation. Sirt1 and PGC-1α promote mitochondrial biogenesis. MiR-222 and miR-17-3p have also been shown to regulate the cell cycle and cardiomyocyte proliferation. Akt, RAC-α serine/threonine-protein kinase; C/EBPβ, CCAAT/enhancer-binding protein-β; CITED4, CBP/p300-interacting transactivator with ED-rich carboxy-terminal domain-4; eIF4E, translation initiation factor 4E; IGF-1, insulin growth factor-1; mTOR, mammalian target of rapamycin; PDK1, phosphoinositide-dependent protein kinase-1; PGC-1α, peroxisome proliferator-activated receptor-γ coactivator 1α; PI3K, phosphoinositide 3-kinase; Sirt1, NAD-dependent deacetylase sirtuin1; S6K1, ribosomal protein S6 kinase-β1; 4EBP1, eIF4E-binding protein 1.

Although human experiments can provide valuable evidence of how exercise influences cardiac regeneration, the limited ability to acquire cardiac tissue is a significant obstacle to exploring the molecular mechanisms of exercise-induced cardiac regeneration. Many studies have shown that the limited capacity of cardiomyocytes to regenerate is enhanced by endurance exercises, such as running and swimming [25,52]. As the most commonly used animal models, rodents (especially mice and rats) have four-chambered hearts that share up to 94% of DNA with humans [53]. Due to their relatively short lifespans, genetic operability, and similar physiological cardiac responses as humans, rodent models are particularly suitable for preclinical study [54,55]. Exercise programs designed for rodents to mimic the effects of exercise on humans include three types of chronic aerobic models, treadmill running, voluntary freewheel running, and swim training [56]. In the running exercise model, exercise intensity is adjusted by controlling the speed, inclination, duration, and interval of the treadmill. The modulation of intensity in the rodent swimming exercise model is changed by the weight of tail loading and exercise duration. The intervention of multiple exercise methods provides a reproducible increase in heart weights from 12–29% and an increase in cardiomyocyte dimension from 17–32% in rodents [57]. Therefore, rodent models offer a valid tool for studying the mechanisms of exercise-induced cardiac regeneration. At present, the mechanism of aerobic exercise-induced myocardial regeneration has mainly focused on paracrine factors, signaling pathways, and miRNAs (Figure 2, Table 1). Therefore, in this section, we explore the relationship between aerobic exercise and the molecular mechanisms of cardiomyocyte regeneration.

### 4.1. Paracrine Factors

Regarding paracrine factors, insulin-like growth factor-1 (IGF-1) and neuregulin-1 have a specific effect on exercise-induced cardiac regeneration. IGF-1, as a growth factor, is released in response to exercise in animal models and elite athletes [58,59]. Compared with sedentary individuals, increased cardiac IGF-1 is related to heart physiologic growth in trained competitive soccer players [60]. IGF-1 binds to IGF-1 receptor (IGF-1R) and mediates exercise-induced cardiac physiological hypertrophy and cardiomyocyte proliferation by initiating a nexus of intracellular signaling [61,62]. In addition, IGF-1R has also been confirmed to be both necessary and sufficient to mediate physiologic growth in mice [63,64]. Moreover, IGF-1R and insulin receptor (IR) control the cardiac metabolic adjustment to exercise. For example, cardiac-specific IR-deletion in mice increases oxidative stress and mitochondrial energetic impairment [65]. Insulin receptor substrate (IRS) deletion attenuates exercise-induced heart growth and metabolic adaption [66]. The role of IGF-1 up- and down-stream regulators has also been investigated. Notably, phosphoinositide 3-kinase (PI3K)/Akt is the critical pathway that transmits IGF-1 signaling in the context of exercise-induced cardiomyocyte regeneration and heart growth [67]. This will be discussed in detail in the following sections.

Neuregulin-1 stimulates the intracellular PI3K signaling pathway by activating ErbB2/ErbB4 tyrosine kinase receptors. Of note, the neuregulin-1/ErbB2/ErbB4 signaling pathway is essential for cardiomyocyte proliferation and differentiation during development [68]. Accompanied by karyokinesis and cytokinesis, the specific action of neuregulin-1 triggers cardiomyocytes to activate the cell cycle in the S phase, so neuregulin-1 is directly involved in proliferation via cell division in adult cardiomyocytes. Hence, the neuregulin-1/ErbB4 and ErbB2 axes are novel genetic and molecular targets for heart regeneration [69]. Exercise training initiates BrdU- and Ki67-positive cardiomyocyte formation by significantly increasing the expression of IGF-1 and neuregulin-1 in rats [52]. Four weeks of running enhances neuregulin-1 concentrations and stimulates ErbB2, ErbB4, and PI3K/Akt signaling to activate cardiomyocyte regeneration in MI rats [26]. CCAAT/enhancer-binding protein-β (C/EBPβ) is a crucial functional target downstream of the ErbB4 signaling pathway, especially of Akt1 in the heart. As a member of the bHLH gene family of DNA-binding transcription factors, C/EBPβ plays an essential role in cell proliferation and differentiation through cell cycle regulation [70]. C/EBPβ expression is decreased in cardiomyocytes following two weeks of endurance swimming. Interestingly, knockdown of C/EBPβ leads to cardiomyocyte proliferation and gene activation, similar to what is induced by exercise [19]. Strikingly, BrdU-positive cardiomyocytes are increased in exercised and C/EBPβ-downregulated mice. The downregulation of C/EBPβ promotes cardiomyocyte proliferation through the negative regulation of CBP/p300-interacting transactivator with ED-rich carboxy-terminal domain-4 (CITED4), which increases the essential cell cycle/proliferation factor cyclinD1 [19]. However, the specific roles for IGF-1 and neuregulin-1 signaling in exercise-induced cardiomyocyte regeneration need further study.

### 4.2. PI3K/Akt/Mammalian Target of Rapamycin (mTOR) Signaling Pathway

Many studies based on transgenic mouse models have found that the PI3K-Akt axis plays a vital role in the process of exercise-induced myocardial regeneration [46,71,72,73]. The heterodimeric kinase PI3K is situated in the plasma membrane, and it catalyzes phosphatidylinositol-3,4,5-trisphosphate (PtdIns (3, 4, 5) P_3_) release. PtdIns (3, 4, 5) P_3_ is inactivated by lipid phosphatases and tensin homolog (PTEN), which is also an endogenous PI3K inhibitor. Regulatory subunits (p85α, p50α) and catalytic subunits (p110α, p110β, or p110δ) comprise PI3K [67,74]. The overexpression of PI3K (p110α) promoter causes physiological growth but no pathologic hypertrophic growth of the heart in mice [64,67], while dominant-negative PI3K (p110α) transgenic mice do not show physiological hypertrophy stimulated by exercise [67]. Akt, a serine/threonine-protein kinase, is activated by PtdIns (3, 4, 5) P_3_ and PI3K-dependent PDK1 phosphorylation [75] in normal conditions. Interestingly, Akt1-deletion mice exhibit impaired cardiac growth after 20 days of swimming training but display hypertrophy in the context of aortic banding [71]. As a driver, the PI3K/Akt signaling pathway is involved in cell cycle regulation [76]. Akt has a favorable regulatory effect on the cell cycle by extending the half-life of cyclin D while inhibiting cyclin D1 degradation of PI3K [77].

The mTOR comprises two distinct serine/threonine kinases, mTOR complex 1 and 2 (mTORC1 and mTORC2, respectively), in which the former is sensitive to rapamycin. The Akt/mTOR pathway is activated following exercise training and is a crucial regulator for cardiac physiological growth [75]. Pharmacologic inhibition of mTORC1 with rapamycin can reverse Akt1 overexpression-induced cardiac hypertrophy [78]. A recent study showed that eight weeks of swimming exercise coupled with growth hormone application increases mTOR protein expression levels in the left ventricular tissue of rats [46]. In addition, S6K1 and 4EBP1, mTOR signaling downstream molecules, act as the crucial factors in governing protein biosynthesis in the process of cardiac growth. Swimming exercise in rats for eight weeks induces left ventricular hypertrophy by regulating the gene expression of specific miRNAs that target the PIK3/Akt/mTOR signaling pathway and its negative regulators [72].

### 4.3. NAD-Dependent Deacetylase Sirtuin1 (Sirt1)/PGC-1α/Akt Signaling Pathway

Mitochondrial functions are adapted to increased energy demand during long-term exercise [79]. Sirt1 is a critical enzyme regulating mitochondrial biogenesis and oxidative stress [80]. Moderate long-term exercise significantly enhances Sirt1 expression and activates its downstream targets, including peroxisome proliferator-activated receptor γ coactivator 1α (PGC-1α) and Akt signaling [81]. PGC-1α is a transcriptional coactivator of nuclear receptors initially recognized as a cold-inducible thermogenic regulator involved in mitochondrial biogenesis in brown fat tissues and skeletal muscle [82]. PGC-1α and PGC-1β-deficient mice have altered mitochondrial function and diminished exercise performance via changes in mitochondrial dynamics and oxidative capacity [83,84]. In skeletal muscle, PGC-1 has been shown to mediate mitochondrial adaptations in response to exercise. Acute exercise training significantly increases PGC-1 transcription and mRNA content in the human vastus lateralis muscle [85]. PGC-1α is abundant in the heart after exercise. It plays an essential role in promoting the enhancement in mitochondrial density that increases the efficiency of ATP production through reduced respiratory activity in cardiac myocytes following exercise [66,86,87]. Cardiac PGC-1 plays versatile functions by directly interacting with and activating the downstream cascade of transcription factors [88]. For example, PGC-1 combines with peroxisome proliferator-activated receptor α (PPARα) to adjust fatty acid import, storage, and oxidation in the heart [89,90]. Swimming [91] and running [92] exercise both increase the expression of PGC-1α of the myocardia of mice. HF is related to the downregulation of PGC-1α gene expression [93]. The interconnecting relationship of AMPK-Sirt1 and PGC-1α helps to regulate cardiomyocyte mitochondrial metabolism. Treadmill running promotes AMPK/PGC-1α signal transduction to decrease reactive oxygen species accumulation in rat myocardium [87]. Four weeks of running training can promote the Sirt1/PGC-1α/Akt signaling pathway in cardiomyocytes of MI rats [94]. Although long-term aerobic exercise can enhance resistance against oxidative stress [95,96], there is still no direct evidence in a heart-specific PGC-1-deficient animal model to confirm the necessary role of PGC-1 in the exercise-induced oxidative stress response and cardiomyocyte regeneration.

### 4.4. miRNAs

miRNAs are small, single-stranded, extremely conserved noncoding RNAs of 21–22 nucleotides that are tightly linked to cardiac disorders. In sports, miRNAs play various roles in exercise-induced cardiomyocyte proliferation and are essential for protecting against pathological cardiac remodeling [20,97,98]. miR-222 is a critical regulator in exercise-induced cardiac regeneration [25]. Swimming exercise increases the expression of miR-222, which induces EdU- and Ki67-positive cardiomyocyte enhancement. In vitro, miR-222 induces a physiologic growth phenotype in cardiomyocytes along with an increased α-MHC/β-MHC ratio and suppresses fetal gene markers like ANF and BNP. MiR-222 also plays a critical role in exercise-induced cardiomyocyte growth after ischemic injury in mice. This effect is mostly mediated by the cell cycle inhibitor p27. Inhibiting the function of miR-222 leads to inhibition of heart growth after exercise [20]. Applying a similar exercise scheme, Shi et al. found that three weeks of endurance exercise increased miR-17-3p, which belongs to the miR-17-92 cluster. TIMP-3 is a target gene of miR-17-3p and indirectly inhibits PTEN. Inhibition of miR-17-3p can attenuate exercise-induced cardiac growth in vivo [97].

Exosomes are small (30–100 nm) endogenous membrane vesicles secreted by most cell types that play a crucial regulatory role in mediating cell-to-cell communication and crosstalk between organs. Exosomes might carry cardioprotective factors induced by remote MI. They also have a favorable effect on oxidative stress following an ischemia-reperfusion injury to the heart. Four weeks of swimming exercise can increase exosomal miR-342-5p, a major cardioprotective factor that inhibits apoptotic signaling (caspase 9 and Jnk2) and enhances survival signaling (p-Akt) in ischemic/reperfused hearts. This has also been confirmed in rowing-trained athletes [99]. Furthermore, ample evidence has confirmed that miRNAs play a vital role in cardiomyocyte regeneration [100,101], which may be related to exercise.

Thus, numerous studies have shown that aerobic exercise can induce the molecular pathways related to cardiomyocyte regeneration, but current studies still lack direct histological evidence. Moreover, further research needs to define the intensity, frequency, and duration of aerobic exercise and explore the precise mechanisms of cell cycle regulation, Wnt/β-catenin signaling [102], the Notch pathway [103], Hippo signaling [104], major paracrine factors, and transcriptional factors on cardiac regeneration.

## 5. Potential for Exercise to Trigger Cardiac Regeneration in Humans

As highlighted in this review, animal models of exercise training have indicated that training can play a regulatory role in cardiac regeneration. However, whether the same outcomes will occur from exercise in humans has yet to be illuminated. A large body of work has shown that aerobic training and resistance training can increase IGF-1 serum concentrations in human subjects [105,106]. Numerous studies have suggested that cardiovascular adaptation occurs following prolonged exercise, including a sustained increase in cardiac output and a 10–20% increase in cardiac dimensions [16]. Although there are methodological difficulties in assessing cardiomyocyte regeneration and renewal in humans, researchers have developed an elegant approach to label newly formed cardiomyocytes in mice. Studies have shown that the renewal rate of cardiomyocytes is around 0.5–1% yearly during an entire human lifespan through ^14^C labeling. In comparison, the cardiomyocyte production rate is around 0.76% per year in mice, as determined by ^15^N-thymidine tracing [13,25]. The cardiomyocyte renewal rate has been found to be even higher after MI. Based on existing research, more experimental data are needed to confirm the effect and molecular mechanisms of exercise on human myocardial regeneration. Thus, with the continuous development of research technology, it is possible to activate and harness the regenerative potential of the human heart through effective exercise methods.

## 6. Summary and Future Perspectives

At present, the overwhelming majority of studies support the use of aerobic exercise as a useful intervention to promote cardiomyocyte regeneration under physiological and pathological settings. Among the complex molecular and cellular mechanisms, IGF-1 and neuregulin-1 are the main paracrine factors, the IGF-1/PI3K/Akt axis is the primary signaling pathway, and miR-222 is the main miRNA regulatory factor in aerobic exercise-induced cardiomyocyte regeneration. Although the relative contribution of aerobic exercise-induced cardiomyocyte regeneration is far from clear, some evidence indicates that cardiomyocyte renewal is necessary for mediating the beneficial effect of exercise against cardiac injury. However, translating experimental findings into therapeutic regimens still requires much work to validate their safety and applicability for clinical use.

Although regular and appropriate exercise plays beneficial role in cardiovascular function, prolonged endurance exercise leading to repeated overstimulation and injury might cause inflammation and fibrosis of the atrial, ventricular septum, and right ventricle, which is the basis of atrial and ventricular arrhythmias [107]. Despite aerobic exercise having positive effects on cardiomyocyte regeneration, the unresolved problems related to exercise type, intensity, and duration require further attention. Moreover, is there a specific signaling pathway that causes exercise adaptation in the heart? If so, is it possible to therapeutically stimulate specific signaling pathways to mimic exercise stimulation? In addition, can exercise-induced neonatal cardiomyocytes and the original cardiomyocytes form regular electrical coupling activity restore the contractile activity of a damaged myocardium, thereby promoting the recovery of cardiac function? The underlying mechanisms of exercise-induced cardiac regeneration should be intensively investigated to generate new functional myocardium and improve cardiac function in injured and diseased hearts. Furthermore, cardiomyocyte hypertrophy partly be a compensatory reaction to declining regenerative capability, which seems insufficient counteract an accumulated loss of myocytes with normal aging [7,47]. Exercise significantly attenuated the age-induced increase of apoptosis through ErbB family of tyrosine kinases in cardiomyocytes [108]. Multiple experiment data also indicated that aerobic exercise improved survival of aged cardiomyocytes by increasing Akt activity in senescent rodent hearts [109,110]. Thus, exercise could effectively regulate cardiomyocytes proliferation to resist age-related hypertrophic and apoptosis in heart. Finally, the intervention of exercise as a therapeutic strategy to promote cardiomyocyte regeneration may be affected by numerous factors, including patient condition and exercise program. Therefore, sports medicine experts and clinicians should formulate individualized exercise programs and establish effective evaluation systems. This will provide a strong foundation for the use of exercise as a useful method to promote cardiomyocyte regeneration in patients.

## Figures and Tables

**Table 1:** Factors related to aerobic exercise-induced cardiac regeneration.

Classification	Factor	Aerobic Exercise Model	Species	Duration	Regenerative Label	Observation	Reference No.
Paracrine factor	IGF-1	Swimming exercise: ramp protocol started from 10 min to 90 min, with 20 min increased each day, twice/day	Mice	4 weeks 7 days/week	None	IGF-1R- and IR-mediated signals in the development of exercise-induced physiological cardiac hypertrophy	[61]
	IGF-1R	Swimming exercise: ramp protocol started from 10 min to 90 min, with 10 min increased each day, twice/day	Mice	5 weeks 7 days/week	None	Cardiac hypertrophy growth induced by exercise blunted in IGF-1R KO mice	[63]
	IGF-1, Neuregulin 1	Running exercise: low intensity (55–60% of individual VO_2max_) and high intensity (85–90% of	Rat	4 weeks 4 days/week	BrdU, Ki67	IGF-1, Neuregulin 1, TGF-β1 ↑ Newly formed cardiomyocytes ↑	[52]
	Neuregulin 1/ErbB/PI3K	Running exercise: ramp protocol started from 10 m/min for 10 min, progressively increased to 16 m/min, 50 min/day	Rat	4 weeks 5 days/week	BrdU, PCNA	Neuregulin 1 expression ↑ Activity of ErbB2, ErbB4, and PI3K/Akt pathway ↑	[26]
	CITED4, C/EBPβ	Swimming exercise: ramp protocol started from 10 min to 90 min, with 10 min increased each day, twice/day	Mice	2 weeks 7 days/week	BrdU, Ki67, AuroraB, pH3	C/EBPβ expression ↓ CITED4 expression ↑	[19]
Signaling pathway	PI3K/Akt/mTOR	Swimming exercise: ramp protocol followed by 60 min sessions with 5% body overload, twice/day	Rat (Female)	8 weeks 5 times/week	None	PIK3/Akt/mTOR pathway gene expression ↑ PTEN gene expression ↓	[72]
	Akt-1	Swimming exercise: each session lasted 90 min, twice/day	Mice	4 weeks 5 days/week	None	Exercise-induced hypertrophy attenuated in Akt1-/- mice	[71]
	mTOR/p70S6K	Swimming exercise: each session lasted 60 min, with 5% body overload, twice/day	Rat (obese)	12 weeks 5 days/week	None	Activity of mTOR/p70S6k pathway ↑	[73]
	Sirt1/PGC-1α	Running exercise: endurance training ramp protocol increased from 4.2 m/min up to 12 m/min for 30 min/day	Rat	36 weeks 4–5 days/week	None	Sirt1 and PGC-1α protein expression ↑	[81]
	Sirt1/PGC-1α/Akt	Running exercise: ramp protocol started from 10 m/min for 30 min/day, progressively increased to 16 m/min, 60 min/day	Rat	4 weeks 7 days/week	None	Sirt1/PGC-1α/PI3K/Akt pathway ↑	[94]
microRNA	miR-222	Running exercise: voluntary wheel running [20,25] Swimming exercise: ramp protocol followed by 90 min, twice/day [20]	Mice	4 weeks 7 days/week [20] 8 weeks 5 days/week [25]	EdU, Ki67, pH3 [20] ^15^N-thymidinel [25]	miR-222 expression ↑ Inhibition of miR-222 blocks the cardiomyogenic exercise response	[20,25]
	miR-17-3p	Swimming exercise: ramp protocol followed by 90 min, twice/day for 3 weeks Running exercise: voluntary wheel running for 3 weeks	Mice	3 weeks 7 days/week	EdU, Ki67, pH3	Inhibition of miR-17-3p attenuates exercise-induced cardiac growth in vivo; Mice injected with miR-17-3p agonist are protected from adverse remodeling after cardiac ischemia/reperfusion injury	[97]

BrdU, 5-bromo-2′-deoxyuridine; C/EBPβ, CCAAT/enhancer-binding protein-β; CITED4, carboxy-terminal domain-4; EdU, 5-ethynyl-2′-deoxyuridine; IGF-1, insulin-like growth factor-1; IGF-1R, IGF-1 receptor; IR, insulin receptor; KO, knockout; miR-17-3p, microRNAs 17-3p; miR-222, microRNAs 222; mTOR, mammalian target of rapamycin; p70S6k, phosphorylation of the 70-kDa S6 protein kinase; PGC-1α, peroxisome proliferator-activated receptor-γ coactivator 1α; PI3K, phosphoinositide 3-kinase; Sirt1, NAD-dependent deacetylase sirtuin1; TGF-β1, transforming growth factor β1; PCNA, proliferating cell nuclear antigen; pH3, phosphor-histone3. ↑, upregulation; ↓, downregulation.

## Data Availability

Not applicable.
